# Intergenerational effects of child maltreatment on adolescents’ anxiety and depression in Ethiopia: the important mediating and moderating roles of current psychological distress

**DOI:** 10.1186/s12888-024-05586-6

**Published:** 2024-02-15

**Authors:** Amare Misganaw Mihret, Nina Heinrichs

**Affiliations:** 1 Clinical Psychology and Psychotherapy, Universität Bremen, Grazer Straße 6, 28359 Bremen, Germany; 2 Department of Psychology, Clinical Child and Adolescent Psychology and Psychotherapy, Bielefeld University Universität Bielefeld, Universität Straße 25, 33615 Bielefeld, Germany

**Keywords:** History of child abuse and neglect, Child maltreatment, Intergenerational associations, Anxiety and depression, Psychological distress

## Abstract

**Background:**

Child abuse is widespread around the world, and one continent with particularly high rates is Africa. Research in high- and middle-income countries shows the cascading effect of parental history of child abuse and neglect on adolescents’ maltreatment and, in turn, on mental health problems. This cascade has been reported in young children but has rarely been studied in parent-adolescent dyads or in low-income countries (LICs). The goal of this study was to test intergenerational associations of child abuse and neglect and to examine how these experiences are in turn associated with youth anxiety and depression in an LIC.

**Methods:**

A total of 231 adolescents (age: 13–21 years) and 185 of their parents (*n* = 90 fathers and *n* = 95 mothers) were recruited from secondary schools in Addis Abeba, Ethiopia. Using a cross-sectional design, participants completed a set of questionnaires assessing child maltreatment (in adolescence and own past history in parents), parental psychological distress, youth depression and anxiety, and sociodemographic factors.

**Results:**

The frequencies of child maltreatment exposure were 68% for adolescents and 65% for their parents (when they were a child). Fifty-one percent and 42% of adolescents had borderline to clinical levels of anxiety and depression symptoms, respectively. Adolescents of parents with a history of child abuse and neglect also reported higher exposure to maltreatment themselves (*p* < 0.001). Current paternal, but not maternal, psychological distress mediated this intergenerational association of maltreatment experiences (95% CI [1.164, 9.467]). We further found parents’ psychological distress to be a significant moderator of the indirect pathways of the intergenerational effect of child maltreatment on adolescents’ anxiety and depression (95% CI [− 0.770, − 0.012]).

**Conclusions:**

We found child maltreatment to be intergenerationally associated, and this effect subsequently affected adolescents’ anxiety and depression through different pathways supporting the cascading effects across generations. Intervention plans may be effective through an array of possible indirect pathways and encourage the implementation of multiple access points to facilitate change in the lives of affected youth in Africa.

**Supplementary Information:**

The online version contains supplementary material available at 10.1186/s12888-024-05586-6.

## Background

Child maltreatment occurs when parents abuse (physically, emotionally or sexually), neglect, expose the child to partner violence, or allow for commercial abuse or exploitation of children under the age of 18 [1]. When parenting is branded by child abuse and neglect, the developmental pathways of children, as well as their social and emotional development, might be significantly obstructed [2, 3]. As a result, it is critical to assess the magnitude, type, and consequences of child abuse and neglect exposure to implement prevention and support systems in countries.

Globally, child maltreatment has been extensively studied, but a true estimate cannot yet be made. Review studies have provided inconsistent results due to a lack of studies in economically weak countries such as Africa [4, 5]. A review that included 23 studies in Africa, with the majority of studies coming from South Africa, showed a prevalence of lifetime physical abuse ranging from 8 to 45% [6]. Africa consists of 54 sovereign states, and 70% of the data in this review originated from South Africa, where only 4% of the population lives. The sovereign states with the densest population are Nigeria and Ethiopia, accounting for 15 and 9% of the continent’s population, respectively. However, there is only one study from Nigeria but none from Ethiopia published and included in the estimates from these states. A true estimate of child abuse and neglect in Africa can only be achieved if more reports are available from African countries, particularly larger ones, that host many families and make a significant contribution to the African population, such as Ethiopia.

The socioeconomic and political situation in Ethiopia has been described as “the worst humanitarian crisis in decades” [7] , p 1. There have been civil wars, conflicts, droughts, displacements, pandemics, macroeconomic deteriorations, and a campaign to demolish civilian houses that have left hundreds of thousands homeless, just to name a few [7]. highlighted that the current scenario affects 29.4 million people and anticipates that even more children and adolescents will be exposed to child maltreatment than before. In addition, authoritarian parenting practices are the rule in Ethiopia [8], which could also raise the rate of child maltreatment [9].

Previous studies (many of which were university theses or gray literature) conducted in Ethiopia have accordingly already shown high numbers. It was found that physical abuse varied from 64% [10] to 70% [11]; emotional abuse varied from 72% [12] to 83% [11]; IPV varied from 58% [13] to 70% [14]; and child labor ranged from 43% [15] to 79% [16]. These studies are, however, limited in several ways: they used unvalidated assessment tools, observed only selected types of child maltreatment and communicated results in local languages only. There is a dearth of high-quality studies in English for large African countries that have a high number of inhabitants and therefore contribute significantly to the population. We therefore aim in the current study to carefully examine various forms of abuse and neglect together in one study, such as physical abuse, emotional abuse, neglect, witnessing IPV, and child labor in Ethiopia, using a well-validated and frequently implemented assessment tool to collect data and intent to make the results available in English as the main language for international research communication.

### Youth anxiety and depression

Anxiety and depression are two of the most frequent mental disorders among adolescents, with the number of patients increasing by 18.4% between 2005 and 2015 [17]. Anxious people may experience symptoms such as fears and worries, restlessness or agitation, whereas depressed people may have a continuously low mood, loss of interest, and enjoyment [18]. The magnitude of anxiety and depression among youths, particularly in Africa, where the highest population of the age group is located compared to the rest of the world, is not well known [19]. Efforts to describe and analyze the levels and course of depression and anxiety are reflected in a recent systematic review of studies from 2008 to 2020: the identified point prevalence of depression and anxiety was 27 and 30% in Sub-Saharan African adolescents, respectively [20]. Studies from Ethiopia reflected varying rates of anxiety and depression ranging from 8% [21] to 67% [22] and 7% [23] to 41% [22], respectively.

### Child maltreatment links to anxiety and depression

There is plenty of evidence that child maltreatment exposure is associated with adolescents’ anxiety and depression. Reports have shown that 14% of adolescents (10–19 years old) suffer from mental health problems due to childhood adversity, primarily depression and anxiety [24]. Adolescents’ maltreatment exposure further increased the risk of anxiety and depression [25, 26]. However, only a few studies on the relationship between child maltreatment and anxiety and depression in Africa exist, and these seem to indicate that child maltreatment affects anxiety and depression or overall mental health. A study by Gelaye and her associates [27] reported that female undergraduate college students who experienced gender-based physical and sexual abuse were four times more likely to suffer from depression. Girma and his colleagues [28] found in adolescents (mean age approx. 17 years) from five randomly selected schools that child adversity scores increased depression symptoms (assessed with the Patient Health Questionnaire; PHQ); however, how child adversity measures were adapted to the African context is not clear (10-item questionnaire). A study that reported only about neglect (using the complete Adverse Childhood Events (ACE) questionnaire) found that adolescents who experienced parental neglect were three times more likely to suffer from depression [29]. Finally, in another study, physical abuse (OR = 1.4) and emotional abuse (OR = 2.1) were shown to be substantially related to an increased risk of common mental disorders among university students in South Africa [30].

In summary, there is also a lack of studies from Africa focusing on the link between child maltreatment and youth anxiety and depression as a primary research goal. Only little is known about the strength of the associations between these constructs. Although there have been some reports on the associations, child maltreatment was not usually considered the main predictor of anxiety and depression and was not comprehensively measured. The recruited participants were college or university students or older adolescents in a developmental phase of transitioning into adulthood with fewer interactions with parents. Finally, other risks and protective factors for anxiety and depression have usually not been considered in these studies. One such additional risk factor is the parental history of child maltreatment when they were young themselves.

### Intergenerational effect of maltreatment on youth anxiety and depression

Empirical findings on intergenerational continuity of child maltreatment have produced mixed results, with some reporting the continuity of maltreatment [31, 32] and others disputing it [33, 34], arguing that the continuity claims are overstated, given that the majority of maltreated individuals do not commit violence [35], and on the contrary, parents without maltreatment histories also initiate abuse or neglect their children [36, 37]. More importantly, it is noteworthy that the continuity of child abuse and neglect has progressively weakened with time [38] or has resulted in a smaller effect size (i.e., from r = .31 to r = .21) since 1995 [39]. In the same vein, a meta-analysis found modest continuity of child maltreatment [40]. Another important variable to consider in this link may be the current psychological distress a parent is experiencing.

The literature has identified parents’ psychological distress as a risk factor [41], but studies that have tested psychological distress as a linking mechanism echoed mixed results. Williams [42] and Grunsfeld [43] identified parents’ distress as a mechanism for the link between a parent’s child abuse and neglect history and their children’s maltreatment exposure, while this was not supported in [31]. According to Pears and Capaldi [44], however, depression and posttraumatic stress disorder have an interaction effect, such that parents who have experienced high levels of abuse and display high levels of depression and PTSD are less likely to abuse than parents who experienced high levels of abuse but did not exhibit high levels of depression or PTSD. There are good grounds to assume that parental psychological distress could function as both a protective factor in this link or a risk factor. It is important to identify under which condition parental psychological distress plays a role in this link.

Recent studies have shown that intergenerational transmission of child maltreatment experiences eventually leads to mental health problems in the next generation [45, 46]. Many studies have focused on maternal history of child maltreatment, identifying this past experience as a predictor of current child maltreatment, which in turn predicts youth mental health symptoms (e.g., Russotti et al., 2021). Another contributing contextual factor to this cascade that has been discussed is parental mental health. Studies have shown that maternal depression increases the risk of intergenerational transmission of child maltreatment [45, 47], as did high maternal stress [46], whereas [48] found no increased risk with three indicators of maternal psychological functioning (consisting of parenting stress, maternal psychological distress as indicated by the SCL-90 Global Severity Index and Dissociation). There are, however, very few studies that have been conducted examining whether psychological distress moderates the indirect link of the intergenerational effect of child maltreatment on adolescents’ anxiety and depression, and the studies that have been conducted have been based on childhood samples and only focused on mother-child dyads. We therefore aim to recruit a proportional number of fathers to extend our knowledge of the intergenerational cascading effect of child maltreatment on anxiety and depression in adolescents. This is of particular importance in cultural contexts where fathers play a more dominant role in family choice and decision [49] compared to countries in which such decisions are jointly made or are in the hand of the primary caregiver, which is in most studies the mothers. Fathers in a patriarchal social structure, such as in Ethiopia, are considered to be socially distant but are highly involved in the abuse and neglect of their children. A study conducted in Ethiopia showed that fathers perceive child care as being the mother’s responsibility, and they were less engaged in daily care activities due to traditional beliefs and work commitments [50]. However, fathers are perceived to be involved in the perpetration of child abuse [51], as they are often assigned the role of punishing the child.

### Research questions of the present study

The overall goal of our study was to estimate rates of child maltreatment and to explore how adolescents’ child maltreatment experiences are linked to their anxiety and depression symptoms and which roles parents’ own history of child maltreatment experiences and their current psychological distress play in these links. We were specifically interested in physical and emotional abuse consequences. Therefore, this study addressed the following questions. (1) What is the estimated rate and forms of child maltreatment experiences among two generations of adolescents and their parents in Ethiopia? (2) What is the level of anxiety and depression symptoms among adolescents? Finally, this study also examined two mediation and mediated-moderation hypotheses: (3) parents’ current psychological distress mediates the effect of child abuse and neglect on adolescent child maltreatment; (4) parents’ current psychological distress symptoms moderate the indirect effect of parents’ history of child abuse and neglect on adolescents’ anxiety and depression through child maltreatment.

## Methods

A cross-sectional research design was used to collect data from adolescents and their parents for this study. Over the course of 5 weeks in October and November 2022, data were collected.

### Participants

Study participants were adolescents enrolled in secondary schools in Addis Ababa during the school year of 2022/23 and their parents (either their fathers or mothers). The total number of students in private (North West 1181) and public (2265 Bulbula) schools was 3446 (1622 males and 1824 females). To ascertain the minimum required sample size to detect a medium effect (0.15), a power analysis was conducted utilizing G*Power software version 3.1* [52]. This analysis incorporated four predictors, namely, the independent variable (IV), moderator, interaction terms of (IV*moderator), and the mediator, assuming a moderate significance level (α = 0.05), and a high power of 0.95 resulted in 119 participants. Furthermore, a compromise power analysis was conducted to determine the significance level and power, considering a medium effect size (0.015), a sample size of *N* = 184, and an error probability ratio (q = 1). The analysis indicated that the study could achieve a high power of 0.98. Consequently, our target sample size was set at 210 participants, accounting for an anticipated 10% dropout rate or incomplete questionnaires.

To recruit adolescents, we initially compiled lists of students from each school, categorized by grade level and gender. Subsequently, a proportional number of students were selected from each stratum, employing a stratified systematic random sampling approach. Upon establishing the count of participants residing exclusively with either their father or mother, we utilized a simple random lottery method. This lottery method was applied in adherence to the rules of systematic random sampling for the remaining dual-parent households, facilitating the determination of the number of fathers and mothers.

Consequently, mothers were selected for every other adolescent from first to the last in the list, and the remaining half were designated as fathers. This process ensured a well-balanced gender distribution among parents in our sample. However, due to physical constraints preventing some parents from participating, only 185 parents (80% of the adolescent sample) were able to participate in the data collection. Following the exclusion of incomplete questionnaires, the final analysis incorporated data from 207 adolescents and 184 parents.

The mean age of the adolescent participants was 16.53 (SD = 1.75), ranging from 13 to 21. Females constituted 55.07% of the adolescent sample (*n* = 144), and the majority lived with both parents (*n* = 86; 41.55%). Cross-gender distribution among parents revealed that male adolescents had 41 fathers and 44 mothers participating, while female adolescents had 48 fathers and 51 mothers participating (see Supplementary Material A (SM A) for more information). The parents included in the study were evenly distributed between mothers (51.63%) and fathers (48.37%), with only one parent per adolescent participating. Parental participants had a mean age of 48.03 years (SD = 8.20), ranging from 25 to 76 years. A significant portion of parents (61.4%) held a minimum college diploma, with 28.80% having a bachelor’s degree, 10.90% attaining a master’s degree, and 21.74% holding a college diploma. Occupationally, 37% were employed as civil servants, while 33.7% worked as sole proprietors, reflecting in total 70.7% of the participant pool.

### Instruments

Self-reported paper-pencil questionnaires were used to measure variables of interest. The adolescent and parent questionnaire contained three parts: 1. Participants’ sociodemographic characteristics, such as gender, age, grade level/level of education, family structure/parent’s occupation, 2. child maltreatment experiences, 3. anxiety and depression in adolescents, psychological distress in parents. Each measure (described below) was reviewed by six experts for contextual validity beforehand, and another six content experts evaluated them to ensure that the assessment tool was culturally appropriately used in Ethiopia.

Child maltreatment for the index group was measured by the validated ICAST trial [53], a 25-item scale with an eight-point rating scale that consists of five dimensions: physical abuse (α = .88), emotional abuse (α = .81), neglect (α = .79), and witnessing IPV (α = .67) [53]. Sexual abuse was excluded for our purpose. As per the expert’s recommendation, four items about physical abuse and one on neglect were added to the original 19 items to obtain a 24-item scale. In addition, as child labor is one of the challenges adolescents face in developing countries, we included six child labor items developed by Fakunmoju and Bammeke [54]. As a result, the child maltreatment instrument used in this study had 30 items asking adolescents to indicate the frequency with which they experienced the list of actions on an eight-point scale (1 no answer, 2 never in my life, 3 not in the past year but it has happened before, 4 once or twice happened a year, 5 several times a year, 6 about once a month, 6 several times a month, 8 once a week or more often). A higher score indicates a higher level of maltreatment exposure. The factor analysis established the expected five dimensions and explained 77.14% of the total variance. The overall internal reliability was Cronbach’s alpha = .95, with all five dimensions above >.90.

The parents’ history of child abuse and neglect was assessed using 14 items adapted from the Original ICAST-R V.3 (2016). ICAST-R also covers physical abuse, emotional abuse, neglect, and sexual abuse. Consistent with the child maltreatment assessment, the sexual abuse scale was not used in the present study. In addition, one new item as suggested by experts, two items about witnessing IPV [53], and seven items about child labor [54] were also included as additional measures of history of child maltreatment to parallel the child maltreatment assessment. Therefore, the total number of items used to measure adults’ retrospective child maltreatment was 24. It was a “Yes”, “No”, and “cannot remember” response item. The internal consistency of the overall measure of history of child abuse and neglect was KR-20 = .86, where the dimensions ranged from KR-20 = .74 to KR-20 = .88 witnessing IPV except for the child labor dimension, which showed lower levels with KR-20 = .54.

Anxiety and depression in adolescents were assessed using the self-report revised child anxiety and depression scale (RCADS-25) [55]. It is among the recommended lists of mental health outcome measures used for children and adolescents by the International Consortium for Health Outcomes Measurement [56]. In the RCADS-25, 15 items track anxiety symptoms, and 10 items track symptoms of major depression, which can be summarized to compute anxiety, depression, and total internalizing symptoms. The items represent six dimensions: major depression (MDD, α = 0.76), generalized anxiety (GAD, α = 0.80), separation anxiety (SAD, α = 0.78), obsessive-compulsive disorder (OCD, α = 0.71), panic disorder (PD, α = 0.85), and social anxiety disorders (SP, α = 0.81). Accordingly, participants were asked how often each item applies to them using a Likert scale (0 = never, 3 = always) [55]. Summing up selected response categories results in total anxiety and depression scores; higher scores indicate higher levels of depression and anxiety. Each test score is transformed into a standard score (T score), and scores less than 65 are categorized as having no anxiety or depression, while scores larger than 65 are indicative of having anxiety or depression (65–69 is borderline clinical threshold or mild depression, and scores greater than 70 are over clinical threshold or severe depression). The factor analysis, as in prior studies, identified six components larger than one eigenvalue explaining a total of 76.78% of the variance, with internal consistency ranging from Cronbach’s alpha = .72 to α = .94 in the present study.

Current psychological distress in parents was measured using the 10-item Psychological Distress Scale [57]. This is a 5-point Likert scale ranging from 1 (never) to 5 (always). The items ask how often participants felt nervous, hopeless, restless or fidgety, depressed like everything was an effort, and worthless in the past 30 days. The total score ranged from 10 to 50, with higher scores representing more significant psychological distress. The scale has excellent internal consistency reliability (α = .89 [57];. Similarly, a factor analysis in our study found that a single component with eigenvalues larger than 1 explained 52.92% of the variance, and the internal consistency was Cronbach’s alpha, α = .90.

### Procedure

After obtaining permission from the proprietors of the tests used in this study to modify them to fit the cultural context of the study environment, six experts examined the tests’ face validity and content validity in each evaluation. A qualified translator completed the forward translation from English to Amharic, and the translator’s consistency was reviewed by a forum of specialists, with the first author serving as the host. Afterward, a few samples were collected among adolescents and adults in the research region to help us identify areas where the phrasing needed to be altered. The updated questionnaire was then sent to a language expert to be translated back into English, and one of the researchers and another language expert evaluated its equivalence with the original form. The Ethiopian National Review Board granted ethical approval for this study. Six data collectors were trained for 2 days to increase their understanding of the study’s idea, as well as their abilities to handle privacy and emergency situations. Last, participants who gave us their permission were given questionnaires, and those who preferred interviews and those who were illiterate were interviewed by data collectors while they wrote their written responses to each item.

### Data analysis

SPSS V.28 data analysis software was used to analyze the data. We then used descriptive statistics to summarize the characteristics of the participants and t tests, and chi-square tests were used to test the differences in child maltreatment exposure and mental health outcomes with respect to the participants’ gender. Next, mediation and mediated-moderation analyses were performed using Haye’s process Macro 4.2 with a 10,000-sample percentile bootstrap estimation technique to assess the indirect effect. Thus, we performed process model number four and seven in order to test simple mediation and moderated mediation analyses, respectively. The 10.000 percentile bootstrap process macro model 7 was selected to account for the complexities of our moderated mediation model. This model is well-suited for capturing interaction effects and mediation simultaneously.

## Results

### Adolescents’ child maltreatment exposure rates

As presented in Table 1, the frequency of overall child maltreatment in adolescents was 68%, where 57 (61%) out of 93 male participants and 84 (74%) out of 114 female participants were found to report child maltreatment in their lifetime. The mean scores of males 97.58 (SD = 25.91) and females 105.57 (SD = 27.38) for child maltreatment were significantly different: t _(205)_ = − 2.21, *p* = .03, with Cohen’s d = −.31 indicating a small to medium effect size. Concerning forms of maltreatment, adolescents reported experiencing a relatively large proportion of emotional abuse (67%), followed by witnessing IPV (62%), child labor (59%), neglect (58%), and physical abuse (52%) (see SM B for the forms of child maltreatment among parents and adolescents). The results suggest that more than half of adolescents have experienced all forms of child maltreatment during adolescence or childhood. Among the forms, only females were more exposed to emotional abuse than males (t _(205)_ = − 2.425, *p* = 0.016, with a small to medium Cohen’s d = 0.34). All assessed forms of abuse and neglect are common in adolescents. (see SM C for the main and total effects of gender and family structure on child maltreatment).

**Table 1:** Descriptive statistics of the study variables

Variables	Gender	N	Mean	SD	Mini	Maxi	Frequency Count and percentage				Skewness	Kurtosis	*t*-statistics^a^ or Chi-square^b^
							Yes	%	No	%			
Child Maltreatment	Male Female Total	93 114 207	79.58 105.70 102.05	24.90 27.38 26.54	51 36 36	147 142 147	57 84 141	61.30 73.70 68.10	36 30 66	38.70 28.30 31.90	−0.40	− 0.87	−2.210^a^*
Anxiety and Depression	Male Female Total	93 114 207	31.29 35.37 33.54	13.92 14.05 14.11	5 5 5	61 67 67	49 64 113	52.70 56.10 54.60	44 50 94	47.30 43.90 45.40	0.24	−0.85	−2.086^a^*
Anxiety	Male Female Total	93 114 207	19.34 21.47 20.52	7.76 8.46 8.20	0 0 0	36 39 39	46 60 106	49.50 52.60 51.20	47 54 101	50.50 47.40 48.80	0.15	−0.80	−1.869^a^
Depression	Male Female Total	93 114 207	11.95 13.91 13.03	8.15 7.45 7.81	0 0 0	28 28 28	38 48 86	40.90 42.10 41.50	55 66 121	59.10 57.90 58.50	0.34	−0.85	−1.811^a^
Parents’ CAN history	Male Female Total	89 95 184	- - .65	- - .48	0 0 0	1 1 1	60 59 119	67.40 62.10 64.70	29 36 65	32.60 37.90 35.30	−0.62	−1.63	0.359^b^
Current psychological distress	Male Female Total	89 95 184	24.12	5.89	13	40	38 42 80	44.20 57.30 43.50	51 53 104	55.80 42.70 56.50	0.58	−0.03	1.018^a^

a = t-statistics, b = chi-square, * significant at alpha < 0.05, skewness and kurtosis are presented only for the total scores

### Parents’ child abuse and neglect exposure rates

The frequency of child abuse and neglect history was 65% among parents, with similar rates reported by mothers (62%) and fathers (67%). Parents also reported having been exposed to different forms of maltreatment, including all assessed forms with physical abuse (63%), child labor (54%), emotional abuse (49%), witnessing IPV (48%), and neglect (27%) (see SM B the Chi-square analysis for the forms). The forms of child abuse and neglect history did not vary by gender, Chi^2^ = .359, *p* = .549 (Table 1).

### Adolescents’ anxiety and depression symptom levels

Mean levels and standard deviations for anxiety and depression can be found in Table 1. The mean standard deviation scores of anxiety, depression, and overall anxiety and depression were 20.52 (8.20), 13.03 (7.81), and 33.54 (14.11), respectively. More than 51% of the sample exceeded the cutoff for anxiety and more than 42% for depression (which is according to Ebesutani et al., 2012 [58] T > 65), reflecting generally high rates of borderline to clinical symptom presentations in central Ethiopia (see SM D for more on anxiety and depression results compared to other studies and SM E for the interaction effects of gender and family structure on anxiety and depression).

### Does parents’ current psychological distress (PD) mediate the effect of parental CAN on adolescent child maltreatment?

**Fig. 1:**
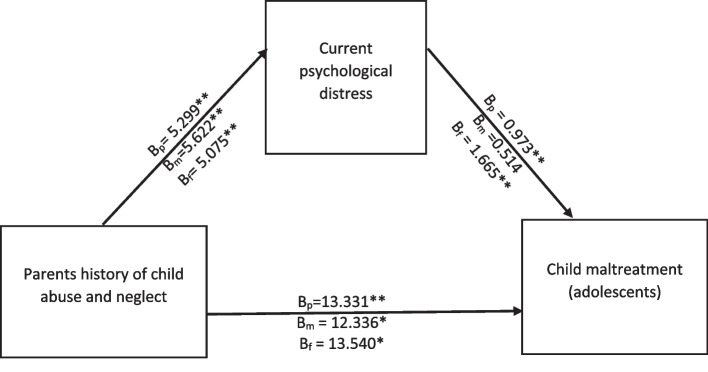
Results of the mediation model. Mediation model number 4 that reveals parents’ history of child abuse and neglect that leads to adolescents’ exposure to child maltreatment directly or through indirect pathways to current parental psychological distress. The same is portrayed from mothers or fathers to adolescents. The beta coefficients of all parents, mothers, and fathers on each path are denoted as B_p_, B_m_, and B_f_, respectively. *p < 0.05, **p < 0.01

After ensuring that the assumptions were not violated, a regression analysis was conducted to examine the intergenerational transmission of child maltreatment across parental and adolescent generations with parental psychological distress, see SM F for parental current psychological distress level comparison to other studies) reflecting the hypothesized mediator of the effect of parental child abuse and neglect history on exposure to adolescent maltreatment (CM, see Fig. 1). We subsequently tested the association for mothers and fathers separately using process macro model four. The results showed that the parents’ child abuse and neglect history was significantly associated with their current psychological distress symptoms (B = 5.299, SE (HC4) = 0.727, 95% CI [3.866, 6.733] β = 0.900, *P* < 0.001) and that the current psychological distress was a significant predictor for adolescent child maltreatment, B = .973, SE(HC4) = 0.401, 95% CI [0.181, 1.764], β = 0.218, *p* = 0.016. The indirect coefficient was significant, B = 5.154, BootSE = 2.148, 95% CI [1.164, 9.467], partially standardized β = 0.196, BootSE = 0.080, 95% CI [0.044, 0.362]. The direct link (parental child abuse and neglect history as a predictor of adolescent child maltreatment) remained significant after mediator control, PD, B = 13.331, SE (HC4) = 4.071, 95% CI [5.298, 21,363], β = .507, *p* = .001, consistent with partial mediation. Furthermore, the total effect was found to be significant at B = 18.484, SE (HC4) = 3.805, 95% CI [10.978, 25.991], β = .700, *p* < .001.

The results demonstrated that the indirect pathway of mothers’ history of child abuse and neglect and adolescents’ child maltreatment did not reach a significant level (b = 2.889, 95% CI -3.668,9.358). However, there was a significant direct effect (B = 12.336, *p* = 0.048) as well as a total effect (B = 15.225, *p* = 0.007). The indirect link from fathers to adolescents, on the other hand, showed a significant effect (B = 8.448, 95CI 3.414,14.466), indicating that adolescent children reported higher child maltreatment exposure via paternal current psychological distress. Furthermore, both the direct relationship (B = 13.540, *p* = 0.011) and the total effect (B = 21.989, *p* > .001) of fathers’ history of abuse and neglect were significant. As a result, fathers’ current psychological distress partially mediated the association of child abuse from fathers to their teens. All regression models can be found in Table 2.

**Table 2:** Results of mediation analysis (parents CAN predict ➔ adolescents’ maltreatment exposure)

Relationship	Total effect	Direct effect	Indirect effect	*t*-statistics	Conclusion
P_CAN➔PD➔A_CM	18.484 (.000)	13.331 (.002)	5.154 (95%CI 2.266,9.795)	2.440	Partial mediation
Mothers CAN ➔mothers PD ➔ Adol_CM	15.225 (.007)	12.336 (.048)	2.889 (95% CI − 3.668,9.358)	0.270	No mediation
Fathers CAN ➔Fathers PD➔ Adol_CM	21.989 (.000)	13.540 (.011)	8.448 (95% CI 3.414,14.466)	2.940	Partial mediation

*CAN* Child abuse and neglect experiences in parents as children, *PD* parental current psychological distress, *CM* child maltreatment experiences of adolescents

### The moderated mediation links of parents’ child abuse and neglect on adolescents’ anxiety and depression

**Fig. 2:**
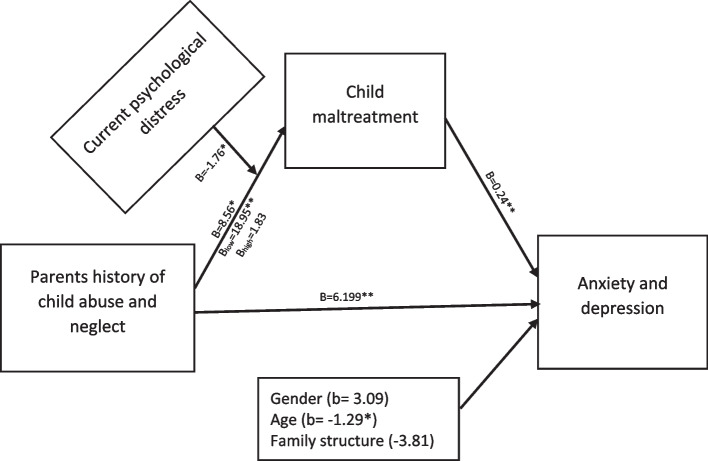
Results of the moderated mediation model. A moderated mediation model number 7 representing the conditional effect of parental child maltreatment history on adolescents’ anxiety and depression through adolescents’ exposure to child maltreatment. The unstandardized beta coefficient (B) is presented in each row. The beta coefficients of different contexts with high, low, and medium levels of psychological distress are referred to as B_high_, B_low_ and B on a-path respectively. *p < 0.05, **p < 0.001

**Fig. 3:**
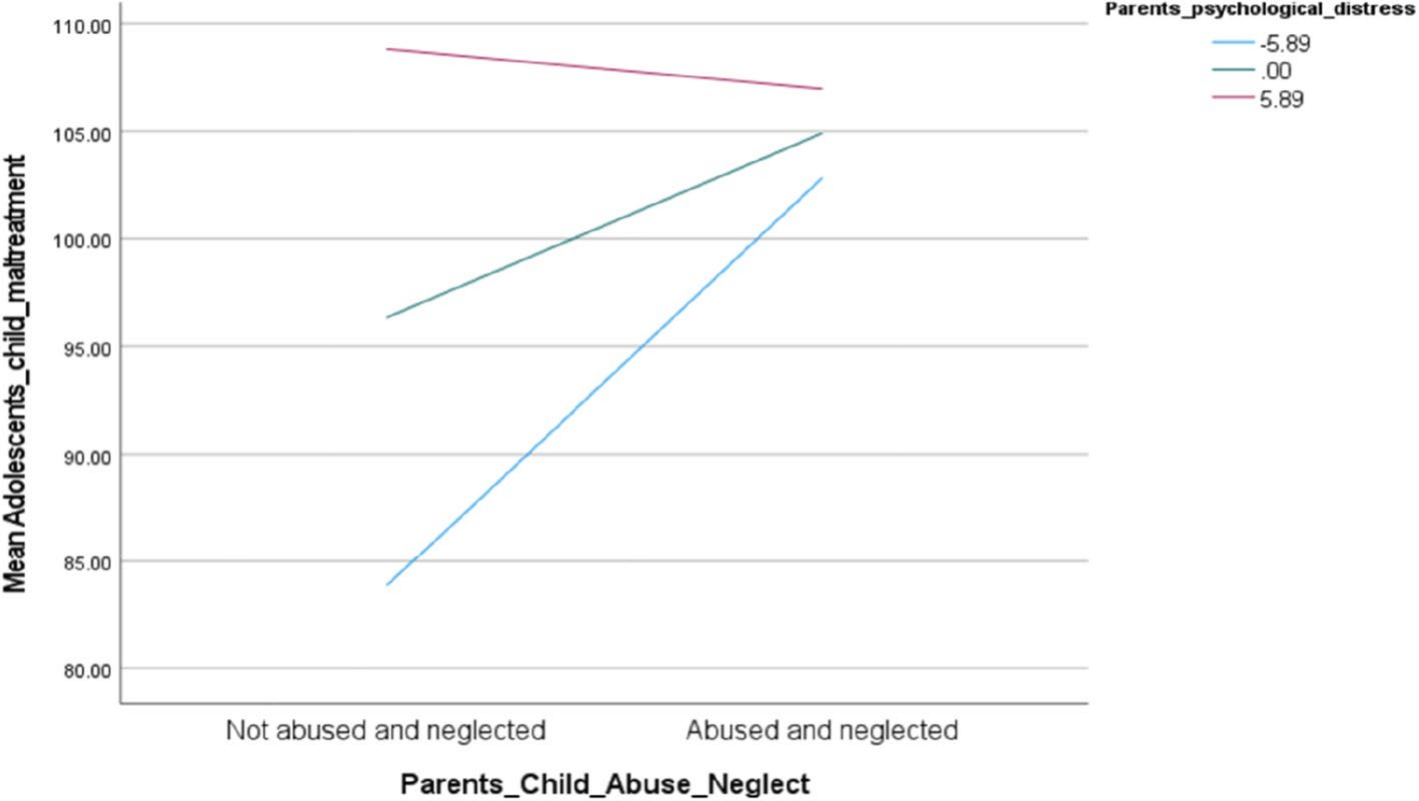
Changes in adolescents’ CM exposure as a function of their parents’ history of CAN. Note: This Figure demonstrates the effect of parents’ history of child abuse and neglect on adolescents’ child maltreatment exposure in the context of high, medium, and low levels of their parents’ current PD symptoms

A regression analysis was carried out to test the hypothesized conditional effect of parents’ PD on the indirect effects of parental child abuse and neglect history on adolescent anxiety and depression via child maltreatment exposure (see Fig. 2) using process macro model number seven. A statistically significant indicator of moderated mediation (B = − 0.416, bootSE = 0.186, 95% CI [− 0.767, − 0.025) was found for parental psychological distress. This was strongest when the parents’ current psychological distress was one standard deviation below the mean (B = 4.471, 95% CI [2.195, 6.922] and somewhat weaker but still significant within less than one standard deviation range (B = 2.020, 95% CI [0.207, 4.385]). No longer significant was the moderation when parents’ current psychological distress was one standard deviation above the mean (B = -0.432, 95% CI [− 3.495, 3.586]). Please refer to Table 3 for the full regression results and the line graph of how the intergenerational association of child maltreatment was contingent on different levels of psychological distress in Fig. 3.

**Table 3:** Direct and indirect effects of the moderated mediation model

Direct relationships/pathways		Unstandardized Coefficient	*t* values	*p* value	
History of CAN ➔ CM (a-path)		8.561	2.092	0.038	
CM ➔ Anxiety & Depression (b-path)		.236	4.977	< 0.001	
History of CAN ➔ Anxiety & Depression (c’-path)		6.199	3.333	0.001	
History of CAN*PD ➔ CM		−1.765	−2.050	0.042	
Conditional indirect effects of Parents history of CAN on Adolescents’ anxiety and depression: Parents’ CAN - > Adolescents Child Maltreatment - > Adolescents’ Anxiety and Depression					
Different levels parents’ current psychological distress	Effect	BootSE	*t* value	BootLLCI	BootULCI
−5.888	4.471	1.215	3.680	2.212	6.961
.000	2.020	1.061	1.904	0.209	4.371
5.888	−0.432	1.793	0.241	−3.477	3.577
Index of moderated mediation:	Index	BootSE	t value	BootLLCI	BootULCI
PD	−.419	.192	2.182	−.770	−.012

*CAN* Child abuse and neglect experiences in parents as children, *PD* parental current psychological distress, *CM* child maltreatment experiences of adolescents

As the number of participants was insufficient to also test parental gender-based continuity of child maltreatment and its subsequent association with anxiety and depression, we only preliminarily explored this (see SM G for full results) and found again that this moderated mediation might be mostly driven by fathers.

## Discussion

We examined intergenerational associations of maltreatment experiences and their links to youth mental health in a unique context. We included several forms of child maltreatment exposure and received valuable information about the frequency estimate of child abuse and neglect covering physical abuse, emotional abuse, neglect, witnessing IPV, and child labor in Ethiopian youth in 2022 (study aim 1). Despite the difficulty of making a comparison of the prevalence rates of child maltreatment studies, given the issue of definition and measurement, we believe the present study at least provides comprehensive information on the distribution in the present sample from Addis Ababa. As in [59], the results of this study revealed a much higher frequency of occurrence of overall child maltreatment in Addis Abeba, Ethiopia, among adolescents (68%) and their parents (65%) than in Western countries. These numbers are even higher than what is reported to be the highest case scenarios of the global prevalence rates, i.e., 50% [4] and 23% [5] for Africa. Similarly, the frequency of maltreatment is markedly higher in our study than in another Ethiopian study on children and adolescents in Gondar Town [13], in which child maltreatment was found to be present in approximately 40% of children and adolescents. This variation could be a result of the measurement of child maltreatment, which was usually less comprehensive than in our study (e.g., 4 items only in Asnake, 2015). In support of this reasoning, a study performed in the same study area that employed a more comprehensive list of physical and emotional abuse items also discovered a much greater prevalence of child maltreatment, similar to our study [11]. The higher rates in our study may also be due to the repeatedly occurring threat of war and violence that swaps from northern and eastern parts of Ethiopia to Addis Abeba.

Findings from Arbamich Town, southern Ethiopia, also indicated that a high proportion of adolescents endured physical punishment [10]. If studies with participants from larger settings of the country were included in the research, the proportion of participants suffering from child abuse increased to more than three-quarters [60, 61]. This may indicate that going further into the countryside (and closer to conflict zones) would increase the likelihood that more adolescents would be exposed to child maltreatment.

Regarding the forms of child maltreatment, the highest frequencies of reported physical abuse, emotional abuse, witnessing IPV, and child labor were found in both groups, parents and adolescents in the present study. However, neglect appeared to be less frequent among parents than adolescents. This variance may result from recall bias [62]. This might also be due to the difficulty of noticing the absence of behaviors in retrospective measures [63] or lifestyle changes experienced by the two generations. One hypothesis may be that the loss of communal culture in the present adolescent generation may have led to a loss of a protective factor against neglect [64, 65] that was still in place with their parent’s generation: Life in Addis Ababa has been massively changed from communal to more individual and reserved and loss of social interdependence since the government’s housing projects were adopted in 2004 [66, 67].

A second aim of this study was to assess the levels of anxiety and depressive disorders in adolescents. These were, similar to the child maltreatment rates, pretty high, with an estimate of 51%. Compared with another recent study in northwestern Ethiopia (67%, [22], our rates were significantly lower (see SM C). This difference could be attributed to the geographical proximity of the participants to the conflict in the region with insurgents of neighboring regions and to the fact that data were collected during COVID-19 in this study. In contrast, lower prevalence rates of anxiety have been reported in a meta-analysis of anxiety disorders in Ethiopia, with an overall pooled rate of 8% from 16 primary studies but with very large ranges from less than 1% up to 63% [21]. Our estimate falls into this range but is on the upper level. In fact, among the five Ethiopian regions covered in the review, Addis Abeba had the highest pooled prevalence (13% instead of 8%). In general, the type of assessment (self-report vs. clinical interview) may have contributed to the large ranges, with self-reports usually yielding higher prevalence rate estimates than clinician-administered interviews [68]. Much lower prevalence rates of anxiety have been reported, with 30% in sub-Saharan Africa [20], 31% in Tanzania [69], 18% in Kenya [70], and 10% in Nigeria [71]. The sociopolitical situation might have contributed to the higher level of anxiety in our sample compared to the Nigerian, Tanzanian and Kenyan samples. In contrast, anxiety was shown to be more prevalent in 66% of secondary school female students in Saudi Arabia [72] and 67% of boarding school students in Malaysia [73]. Various methodological conditions, including data collection measures, sampling, and study areas, may have contributed to these differences. In sum, our results indicate that anxiety symptoms among adolescents were truly high, and even if prevalence rates of associated disorders are likely lower when symptom presentation is followed-up by a clinician, these high screening rates for anxiety disorders call for more attention to the mental health of youth in Ethiopia.

We found similar results for depressive symptoms, with approximately 42% of adolescents in our study reporting high levels of depressive symptoms. Previous studies in Ethiopia reported somewhat lower rates of depression between 22 and 29% [74–76] but also similar rates from 36 to 38% [29, 77]. In general, these rates are quite high and, in line with the anxious symptom reports, require further attention.

Our third aim was to examine parental psychological distress in the link between parents’ own history of child abuse and neglect and the next generations’ experiences in adolescents. The notion of intergenerational continuity of child maltreatment was established in the literature, although some evidence raises questions on the magnitude of effect size. In line with this notion, we found that adolescents of parents with a history of child abuse and neglect reported higher child maltreatment exposure. This finding supports previous findings despite claims that the intergenerational continuity of child maltreatment diminishes over time [36, 63]. Despite the complexity intergenerational transmission of child maltreatment, the presence of a parent’s history of child abuse and neglect should raise concerns about adolescent exposure to these types of conditions in Ethiopia.

In addition, we found that parents’ psychological distress was an important linking mechanism between parents’ and adolescents’ maltreatment exposure. Our study indicated that both mother-to-child and father-to-child transmission of child maltreatment was possible through a direct pathway. It was, however, found that the indirect link through their current psychological distress was not significant for mother-to-child transmission of child maltreatment, while father-to-child was significant. The father-to-child link was in accordance with previous studies that demonstrated that psychological health was one of the factors contributing to the perpetuation of violence [47, 78]. In fact, studies have demonstrated that psychological distress is not associated with intergenerational child maltreatment [48, 79]. Different studies may have assessed psychological distress differently and may put emphasis on specific aspects of this distress. Many studies focusing on depressive symptoms as an indicator of psychological distress found supporting evidence for the link, while others that focused more on general health or on a variety of symptom presentations across domains (not only depression) found less support for this hypothesis. We used the K10, which also taps into depressive symptoms, with some items also covering more general symptoms.

In contrast to our study, most of the previous studies dominantly focused on mother-to-child continuity of child maltreatment and reported maternal psychological distress as a risk factor for intergenerational maltreatment [80, 81]. This is not in line with our finding, and the discrepancy could be attributed to buffering factors such as social support [47], where women are believed to receive more social support than their male counterparts [82]. In Ethiopia, it is widely disseminated that fathers are feared, and they are responsible for disciplining their children, which would in turn lead them to engage in severe disciplinary actions. According to Duindam et al. [83] and in line with the cultural belief, fathers indicated that hitting is a normal and necessary part of upbringing in their perspective. It is also noteworthy that fathers at the same time are less likely to be directly involved in child care tasks in Ethiopia than mothers are [84]. Their assigned role is disciplining and financially saving the family, which leads to work-related absence, which may easily shift to child neglect [85]. Our study, however, was performed with a small number of fathers and mothers if considered separately as well as using data solely from fathers or mothers of adolescent participants, making it difficult to draw on the family as a unit. Child maltreatment in Ethiopia is a phenomenon that evolves along generational lines, and current psychological distress among parents preserves this dysfunctional cycle, especially from fathers to adolescents. This calls for researchers and practitioners to put more emphasis on fathers and their role in child mental health in Ethiopia.

Meanwhile, the complex interplay between parents’ history of abuse and neglect and their current psychological distress, resulting in offspring’s child maltreatment exposure, which subsequently leads to adverse mental health outcomes for adolescents, is difficult to explain by candid underlying relationships. People who have experienced maltreatment during childhood may experience mental health outcomes through a variety of different pathways [86, 87]. Consistent with previous studies, our results highlighted that the child maltreatment experiences of parents and adolescents themselves lead to heightened anxiety and depression symptoms in adolescents. This finding is consistent with previous studies showing that parents’ history of abuse leads to their offspring having worse psychological health outcomes [45, 46].

In addition, we found that the effect of parents’ child abuse history on adolescents’ anxiety and depression through adolescents’ child maltreatment exposure was strongest at low and moderate levels of parents’ current psychological distress. Child maltreatment was more prevalent under these conditions, and eventually, anxiety and depression symptoms also became more prevalent, which is in line with results from other studies demonstrating psychological distress to be a risk factor for the intergenerational cascade of child maltreatment and mental health outcomes [80, 88]. However, the indirect effect of intergenerational child maltreatment exposure on adolescents’ anxiety and depression was not significant at the highest level of parents’ current psychological distress. Correspondingly, there is evidence showing that parents with higher symptoms of PTSD and depression were less likely to abuse their children [44]. On the other hand, there are few findings reflecting that parental psychological distress has no effect on the cascade on intergenerational child maltreatment [48, 79]. The severity of psychological distress has rarely been differentiated and may explain the different results across studies. Engaging in abusive behavior requires a certain level of behavioral action, which might not be possible for parents with more severe depressive symptoms. Another explanation could be that more severe consequences of one’s own history of child maltreatment with posttraumatic stress disorder in adults may protect one from engaging in abusive behaviors toward one’s own children. Finally, a third explanation could be that with severe psychological distress, it does not matter anymore if child maltreatment in the own history occurred or not, it is sufficient to dominate the link and engage in maltreatment of the own child.

In sum, the moderated mediation findings imply that the effect of parental history of abuse and neglect on adolescent anxiety and depression symptoms related to adolescents’ child maltreatment is determined by the severity of psychological distress experienced by the parents. However, further research is needed on the factors involved in the development process of anxiety and depression in adolescents, as the mediators and moderators examined in our study were not able to fully explain the process. We believe that the consideration of additional linking mechanisms such as emotion regulation would contribute to a comprehensive understanding, and risk factors such as the lack of communication in families, chronic child maltreatment, socioeconomic factors, substance abuse, and protective factors such as social support would contribute to a deeper understanding of the intergenerational effects of child abuse and neglect.

## Limitations and strengths

There are a number of limitations to this study, including the cross-sectional design that does not allow for causal inferences, missing a perspective from both parents as we recruited only one of the parents, lower internal consistency of the child labor sub-scale when parents reported about their recollections of their own childhood and the lack of considering other important concepts that are relevant when discussing maltreatment, such as emotion regulation or family and extended social support. There was also inability to achieve full participation from the intended parent sample, resulting in the inclusion of only 80% of the initially targeted parent participants. Consequently, the analysis of adolescents and their parents was based on 89% of the available adolescent data. This limitation may impact the generalizability of the findings and should be considered when interpreting the results. Nevertheless, this is among the first studies that looked at child maltreatment in the second-largest African country, using well-validated and state-of-the-art assessment tools for child maltreatment and youth mental health.

## Conclusion

We established high rates of child maltreatment experiences in both parent and child generations, as well as high rates of anxiety and depression in adolescence. General psychological distress played a crucial role in partially bridging the gap, linking the parents’ history of abuse with the experiences of the adolescent generation, especially from father to adolescent. Our study showed that adolescents’ anxiety and depression were connected to intergenerationally continued child maltreatment experiences of parents and their teens at various levels of parents’ psychological distress. Therefore, the effects of parental history on adolescents’ mental health, as well as the moderating influence of parental psychological distress and the mediating effect of child maltreatment experiences, should be considered when developing interventions to address adolescent mental health or break the cycle of child maltreatment. However, this study showed that there are additional factors that were not included in this study accounting for the explanation of the association of factors leading to adolescents’ anxiety and depression.

### Supplementary materials

Sociodemographic characteristics of the participants, analysis of additional objectives about the forms of child maltreatment among parents and their teens, interaction effects of gender and family structure on adolescents’ child maltreatment exposure, levels of anxiety and depression in comparison to other studies, interaction effects of gender and family structure on anxiety and depression, levels of parental current psychological distress compared to previous studies, and gender specific cascade of parental history of child abuse and neglect to adolescents’ child maltreatment and in turn to anxiety and depression contingent up on parental current psychological distress were included in the supplementary material.

### Supplementary Information

**Additional file 1.**

## Data Availability

The data utilized in this study are not publicly accessible online; however, interested parties can request access to it from the corresponding author. Furthermore, supplementary data related to this study have been included for reference.

## Abbreviations

ICAST: IPSCAN child abuse screening tool
ICAST-R: IPSCAN child abuse screening tool – retrospective version
ICAST trial: IPSCAN child abuse screening tool – child version validated in the South Africa context
CAN: child abuse and neglect
CM: child maltreatment
PD: psychological distress
K-10: Kessler’s psychological distress scale
RCADS-25: revised child anxiety and depression scale
