# Supplementary material for: Intergenerational effects of child maltreatment on adolescents’ anxiety and depression in Ethiopia: the important mediating and moderating roles of current psychological distress
Source: BMC Psychiatry. 2024 Feb 15;24:126. doi: 10.1186/s12888-024-05586-6 (PMC10870629; doi:10.1186/s12888-024-05586-6)
Supplement: Supplementary file 1 — Additional file 1. [file 12888_2024_5586_MOESM1_ESM.zip › Supplementary/Additional filde_amended_without track changer.docx]

**Supplemental Material to the article submitted on “intergenerational effects of child maltreatment on adolescents’ anxiety and depression in Ethiopia: the important mediating and moderating roles of current psychological distress”.**

Amare Misganaw Mihret^1, 2^ and Nina Heinrichs^1,2^

^1^ University of Bremen, Clinical Psychology and Psychotherapy

^2^Bielefeld University, Department of Psychology, Clinical Child and Adolescent Psychology and Psychotherapy

**Introduction**

The document incorporated in this study constitute supplementary analyses and hypothesis testing which was not included in the main text. We anticipate that these analyses could offer essential insights, enhancing our comprehension of the data's inherent nature and the interactions among variables. The document is structured based on the locations outlined in the main manuscript. For instance, sociodemographic characteristics of the participants as Supplemental Material A (SM A), analysis of different forms of child maltreatment is denoted as SM B, while the main and total effect of gender and family structure is presented as SM C. Levels of anxiety and depression are detailed in SM D, and the primary and interaction effects of gender and family structure on anxiety and depression are covered in SM E. Additionally, SM F addresses the parents' current psychological distress levels, and SM G explores the gender-based transmission of child maltreatment from fathers or mothers to adolescents, subsequently impacting anxiety and depression. The information is presented through tables, descriptions, and, in some cases, discussions, following their respective order."

**Supplemental Material (SM) A**

**Sociodemographic characteristics of the participants**

**SM A Table 1.**

**Gender-based distribution of the participants sociodemographic characteristics**

| **Variables** | | **Male** | | **Female** | | **Total** | |
| --- | --- | --- | --- | --- | --- | --- | --- |
|  |  | **N** | **%** | **N** | **%** | **N** | **%** |
| Parents | Fathers | 41 | 48.2 | 48 | 48.5 | 89 | 48.4 |
|  | Mothers | 44 | 51.8 | 51 | 51.5 | 95 | 51.6 |
|  | Total | 85 | 100 | 99 | 100 | 184 | 100 |
| Grade Level (adolescents) | 9^th^ grade | 29 | 14.01 | 51 | 24.64 | 80 | 38.65 |
|  | 10^th^ grade | 23 | 11.11 | 27 | 13.04 | 50 | 24.15 |
|  | 11^th^ grade | 29 | 14.01 | 17 | 8.24 | 46 | 22.22 |
|  | 12^th^ grade | 12 | 5.80 | 19 | 9.18 | 31 | 14.98 |
|  | Total | 93 | 44.93 | 114 | 55.07 | 207 | 100 |
| Family structure | With both parents | 43 | 20.77 | 43 | 20.77 | 86 | 41.55 |
|  | With father only | 4 | 1.93 | 9 | 4.35 | 13 | 6.28 |
|  | With mother only | 17 | 8.24 | 19 | 9.18 | 36 | 17.39 |
|  | With father & stepmother | 8 | 3.86 | 11 | 5.31 | 19 | 9.18 |
|  | With mother & stepfather | 6 | 2.90 | 7 | 3.38 | 13 | 6.28 |
|  | With extended family | 10 | 4.83 | 8 | 3.86 | 18 | 8.70 |
|  | With nonrelative | 3 | 1.45 | 2 | 0.97 | 5 | 2.42 |
|  | Other (by their own, Orphan, employers) | 2 | 0.97 | 15 | 7.26 | 17 | 8.24 |
|  | Total | 93 | 44.93 | 114 | 55.07 | 207 | 100 |
| Level of education (parents) | Illiterate | 0 | 0.00 | 6 | 3.26 | 6 | 6.26 |
|  | Primary school | 12 | 6.52 | 13 | 7.07 | 25 | 13.59 |
|  | Secondary school | 11 | 5.98 | 29 | 15.76 | 40 | 21.74 |
|  | College diploma | 22 | 11.96 | 18 | 9.78 | 40 | 21.74 |
|  | Bachelor’s degree | 32 | 17.39 | 21 | 11.41 | 53 | 28.80 |
|  | Master’s degree & above | 12 | 6.52 | 8 | 4.35 | 20 | 10.87 |
|  | Total | 89 | 48.37 | 95 | 51.63 | 184 | 100 |
| Parents’ occupation | Unemployed | 1 | 0.5 | 22 | 12.0 | 23 | 12.5 |
|  | Daily labor/unskilled work | 1 | 0.5 | 10 | 5.4 | 11 | 6.0 |
|  | Private business / trade | 28 | 15.2 | 34 | 18.5 | 62 | 33.7 |
|  | Civil servants/NGO | 40 | 21.7 | 28 | 15.2 | 68 | 37.0 |
|  | Taxi/driver | 11 | 6.0 | 1 | 0.5 | 12 | 6.5 |
|  | Religious leader | 5 | 2.7 | 0 | 0.0 | 5 | 2.7 |
|  | Other (Retired) | 3 | 1.6 | 0 | 0.0 | 3 | 1.6 |
|  | Total | 89 | 47.3 | 95 | 51.63 | 184 | 100 |

**Supplementary Material (SM) B**

**Frequency of Forms of Child Maltreatment**

As shown in SM B Table 2, adolescents reported experiencing a relatively large proportion of emotional abuse (67.1%) followed by witnessing IPV (62.3%), child labor (58.9%), neglect (58%), and physical abuse (52.2%) in their order. This means that the forms of maltreatment mentioned above are dominantly practiced in the study area. The results suggest that more than half of adolescents have experienced all forms of child maltreatment during adolescence or childhood. As shown in SM A Table 1, emotional abuse (t205=2.436, p<0.016) was the only form of child maltreatment that reached statistically significant difference among male (mean=26.73, SD= 8.04) and female (Mean=29.53, SD =8.41) participants. The result suggested that females were more exposed to emotional abuse than their male counterparts. However, there was no statistically significant difference in scores for physical abuse, neglect, witnessing violence against children, and child labor.

Parents were also found to have been exposed to different forms of child abuse and neglect, including physical abuse (63.04%), child labor (53.83%), emotional abuse (49.46%), witnessing IPV (47.87%), and neglect (26.63%). A Chi-square test (with Yates Continuity Correction) revealed no significant association between gender and all forms of child maltreatment.

SM B Table 2

Gender based distribution of adolescents and their parent’s child maltreatment exposure

| **Variables** | | **Male (89)** | | **Female (95)** | | **Total (184)** | | **Male** | |  |  |  | **Female** |  |  | **T^a^ or Χ^2b^** | **p** |
| --- | --- | --- | --- | --- | --- | --- | --- | --- | --- | --- | --- | --- | --- | --- | --- | --- | --- |
|  |  | **N** | **%** | **N** | **%** | **N** | **%** | **Mean** | **SD** | **Mini.** | **Maxi.** | **Mean** | **SD** | **Mini.** | **Maxi.** |  |  |
| Physical abuse  Adolescents | Not abused | 51 | 54.80 | 48 | 42.10 | 99 | 47.80 | 25.34 | 8.94 | 8 | 48 | 27.74 | 10.21 | 8 | 51 | -1.797^a^ | 0.074 |
|  | Abuse | 42 | 45.20 | 66 | 57.90 | 108 | 52.20 |  |  |  |  |  |  |  |  |  |  |
|  | Total | 93 | 100 | 114 | 100 | 207 | 100 |  |  |  |  |  |  |  |  |  |  |
| Parents | Not abused | 28 | 31.5 | 40 | 42.1 | 68 | 37.0 |  |  |  |  |  |  |  |  | 1.801^b^ | .180 |
|  | Abuse | 61 | 68.5 | 55 | 57.9 | 116 | 63.0 |  |  |  |  |  |  |  |  |  |  |
|  | Total | 89 | 100 | 95 | 100 | 184 | 100 |  |  |  |  |  |  |  |  |  |  |
| Emotional abuse  Adolescents | Not abused | 39 | 41.9 | 29 | 25.4 | 68 | 32.9 | 26.73 | 8.41 | 11 | 43 | 29.53 | 8.04 | 8 | 43 | -2.425^a^ | 0.016 |
|  | Abuse | 54 | 58.1 | 85 | 74.6 | 139 | 67.1 |  |  |  |  |  |  |  |  |  |  |
|  | Total | 93 | 100 | 114 | 100 | 207 | 100 |  |  |  |  |  |  |  |  |  |  |
| Parents | Not abused | 46 | 51.7 | 47 | 49.5 | 93 | 50.5 |  |  |  |  |  |  |  |  | .023^b^ | .879 |
|  | Abuse | 43 | 48.3 | 48 | 50.5 | 91 | 49.5 |  |  |  |  |  |  |  |  |  |  |
|  | Total | 89 | 100 | 95 | 100 | 184 | 100 |  |  |  |  |  |  |  |  |  |  |
| Neglect  Adolescents | Not abused | 44 | 47.3 | 43 | 37.7 | 87 | 42 | 19.32 | 6.90 | 7 | 23 | 20.50 | 7.07 | 6 | 32 | -1.204^a^ | 0.230 |
|  | Abuse | 49 | 52.7 | 71 | 62.3 | 120 | 58 |  |  |  |  |  |  |  |  |  |  |
|  | Total | 93 | 100 | 114 | 100 | 207 | 100 |  |  |  |  |  |  |  |  |  |  |
| Parents | Not abused | 62 | 67.7 | 73 | 76.8 | 135 | 73.4 |  |  |  |  |  |  |  |  | .873^b^ | .350. |
|  | Abuse | 27 | 30.3 | 22 | 23.2 | 49 | 26.6 |  |  |  |  |  |  |  |  |  |  |
|  | Total | 89 | 100 | 95 | 100 | 184 | 100 |  |  |  |  |  |  |  |  |  |  |
| Witnessing IPV  Adolescents | Not abused | 37 | 39.8 | 41 | 36 | 78 | 37.7 | 6.99 | 2.34 | 4 | 11 | 7.30 | 2.29 | 4 | 11 | -0.975^a^ | 0.331 |
|  | Abuse | 56 | 60.2 | 73 | 64 | 129 | 62.3 |  |  |  |  |  |  |  |  |  |  |
|  | Total | 93 | 100 | 114 | 100 | 207 | 100 |  |  |  |  |  |  |  |  |  |  |
| Parents | Not abused | 42 | 47.2 | 54 | 56.8 | 96 | 52.2 |  |  |  |  |  |  |  |  | 1.350^b^ | .245 |
|  | Abuse | 47 | 52.8 | 41 | 43.2 | 88 | 47.8 |  |  |  |  |  |  |  |  |  |  |
|  | Total | 89 | 100 | 95 | 100 | 184 | 100 |  |  |  |  |  |  |  |  |  |  |
| Child labor  Adolescents | Not abused | 42 | 45.2 | 43 | 37.7 | 85 | 41.1 | 19.19 | 5.69 | 12 | 28 | 20.64 | 6.31 | 6 | 29 | -1.713^a^ | 0.088 |
|  | Abused | 51 | 54.8 | 71 | 62.3 | 122 | 58.9 |  |  |  |  |  |  |  |  |  |  |
|  | Total | 93 | 100 | 114 | 100 | 207 | 100 |  |  |  |  |  |  |  |  |  |  |
| Parents | Not abused | 39 | 43.8 | 46 | 48.4 | 85 | 46.2 |  |  |  |  |  |  |  |  | .228^b^ | .633 |
|  | Abused | 50 | 46.2 | 49 | 51.6 | 99 | 53.8 |  |  |  |  |  |  |  |  |  |  |
|  | Total | 89 | 100 | 95 | 100 | 184 | 100 |  |  |  |  |  |  |  |  |  |  |

a = t-statistics, DF = 205; b= chi-square results

**Supplemental Material (SM) C**

**Do Gender and Family Structure Produce Significant Main and Interaction Effects on Adolescents’ Child Maltreatment Exposure?**

A statistically significant main effect was observed for family structure: F (1,203) = 24.597, p= .001; with more child maltreatment exposure occurring in non-intact families compared to intact families. However, the effect size was medium (partial eta square η²= 0.108). No further main effects (gender) or interaction effects were found (main effect gender: F (1, 203) =3.359, p= .068; interaction effect gender*family structure, F (1,203) = .059, p =.808).

Regarding adolescents’ exposure to child maltreatment, the literature reflects mixed results when it comes to the effect of gender on adolescents' experiences of child abuse and neglect. It ranges from no effect (1,2) to males reporting more child maltreatment (3–5) and up to females reporting more of these experiences than males (6,7). It should be noted, however, that the gender differences reported in those studies were not the result of similar forms of abuse or neglect; in fact, when sexual abuse was included in the study, it appears that the females were more likely to outweigh the males. In this sense, there was evidence that showed males were prone to physical abuse and females to sexual abuse (8), and sexual and emotional abuse (6). Our study revealed that females' emotional abuse and general child maltreatment exposure were significantly higher than their male counterparts. A stringent analysis of variance, however, did not detect gender as a main effect due to the smaller variance.

*SM C Table 3:*

*Males’ and Females’ Child Maltreatment Scores by Family Structures* and the ANOVA Summary of the Effects of Family Structures and Gender on Adolescents’ CM Exposure

| **Variables** | **Child maltreatment** | | | | | |  | **Type II sum of squares** | **df** | | **Mean square** | **F** | **Sig.** | **Partial Eta Squares** |
| --- | --- | --- | --- | --- | --- | --- | --- | --- | --- | --- | --- | --- | --- | --- |
| **Gender** | **Intact** | | | **Non-intact** | | | **Source** |  |  | |  |  |  |  |
|  | **N** | **Mean** | **SD** | **N** | **Mean** | **SD** |  |  |  | |  |  |  |  |
| Male | 43 | 88.60 | 25.97 | 50 | 105.30 | 21.33 | Corrected Model | 18903.765^a^ | 3 | | 6301.255 | 10.133 | <.001 | .130 |
| Female | 43 | 94.23 | 23.10 | 71 | 112.65 | 27.57 | Intercept | 1993007.404 | 1 | | 1993007.404 | 3204.937 | <.001 | .940 |
| Total | 86 | 91.42 | 24.60 | 121 | 109.61 | 25.34 | Gender | 2089.073 | 1 | | 2089.073 | 3.359 | .068 | .016 |
|  |  |  |  |  |  |  | Family structure | 15295.519 | 1 | | 15295.519 | 24.597 | <.001 | .108 |
|  |  |  |  |  |  |  | Gender * Fam_stru | 36.706 | 1 | | 36.706 | .059 | .808 | .000 |
|  |  |  |  |  |  |  | Error | 126236.651 | 203 | | 621.855 |  |  |  |
|  |  |  |  |  |  |  | Total | 2301013.000 | 207 | |  |  |  |  |
|  |  |  |  |  |  |  | Corrected Total | 145140.415 | 206 | |  |  |  |  |
| a. R Squared = .130 (Adjusted R Squared = .117) | | | | | | | | | |  |  |  |  |  |

Finally, we found a non-intact family environment to be strongly associated with greater exposure to child abuse and neglect. However, contrary to our expectations, this study found no statistically significant interaction effects and gender differences in the participants’ child maltreatment exposure. This could be because the participants were drawn from only urban areas where the majority of the participant’s parents have attained at least a college certificate. This could probably mean that due to the education effect, parents are likely to have relatively balanced expectations of behaviors from (9), and disciplinary measures for males and females. Consistent with this, the existing pieces of evidence showed that rural dwellers (10) and illiterates were reported to have positive attitude towards experiencing physical violence (11,12).

As the non-intact category included single parents, step-parents, other relatives, and non-biological parents, it was our expectation that living with the nonintact families to have a greater risk of child maltreatment. Indeed, individuals from single-parent families were more likely than nuclear families to have been exposed to child maltreatment, according to Asnake (3), which may be due to the crisis faced by the means of becoming to this living arrangement, parental burdens with various obligations, and high expectations of their children (13). According to Gelles (1997), single parents are more likely to engage in abusive conduct, due to the lack of a partner to provide financially, share parenting tasks, and moderate conflicts with children (14). Likewise, step-parents are identified as risk factors for child maltreatment perhaps due to the resistance of parents to build emotional bonds with step-children (15).

**Supplemental Material (SM) D**

**Level of adolescents’ anxiety and depression**

The level of anxiety and depressive disorders observed in the present study were pretty high at 51%. Comparing it with other recent study in north-west Ethiopia at 66% (16), it showed significantly lower level (see SM D Table 4a). This difference could be attributed to the proximity of the participants to the conflict in the region with insurgents of neighboring regions and Covid-19. In contrast, lower prevalence rates of anxiety have been reported in Ethiopia at 8% (17), and 25% (18). It may be due to the fact that Boru and Deressa’a study (17) had high heterogeneity among the included studies, while Gebreegziabher et al. (18) conducted their study in an era when covid-19 was almost forgotten. This indicates, therefore, that anxiety symptoms among our participants were really high, requiring attention from a practitioner in order to prevent them from worsening.

Much lower prevalence rates of anxiety have been reported in 30% in sub-Sahara Africa (19), 31% in Tanzania (20), in Kenya 18% (21), and 10% in Nigeria (22). The socio-political situation might have contributed to the higher level of anxiety in our sample compared to the Nigerian, Tanzanian and Kenyan samples. In contrast, anxiety was shown to be more prevalent in 66% of secondary school female students in Saudi Arabia (23) and 67% of boarding school students in Malaysia (24). Various methodological conditions, including data collection measures, sampling, and study areas, may have contributed to these differences.

Approximately 42% of adolescents in this study were found to suffer depression symptoms. Comparing this result with Northwest and southwest samples where depressive symptoms were reported at 41.4% (16) (SM D Table 4b) and 28% (25) respectively, our sample had significantly higher depressive symptoms than the Southwest sample (SM D Table 4c). Unlike the present findings, the previous studies in Ethiopia reported a lower rate of depression at 28% (18), 29% (26), and 22% (27).

SM D Table 4,

The Level of Adolescents’ Anxiety and Depression

| Variable | Study | | | Age of Sample range, mean & SD | Prevalence rate | | |  | Observed | | | Expected | | Residual | | X^2^ | | | DF |  |
| --- | --- | --- | --- | --- | --- | --- | --- | --- | --- | --- | --- | --- | --- | --- | --- | --- | --- | --- | --- | --- |
| Table 3a |  | | |  |  | | |  |  | | |  | |  | |  | | |  |  |
| Anxiety  No  Yes | Nakie et al. (2022) | | | 15-25, 18.59 & 1.792 | 66.7 | | |  | 101  106 | | | 68.9  138.1 | | 32.1  -32.1 | | 22.368^a^ | | | 1 |  |
| Table 3b |  | | |  |  | | |  |  | | |  | |  | |  | | |  |  |
| Depression  No  Yes | Nakie et al. (2022) | | | 15-25, 18.59 & 1.792 | 41.4 | | |  | 121  86 | | | 121.1  85.9 | | -.1  .1 | | .000^b^ | | | 1 |  |
| Table 3c |  | | |  |  | | |  |  | | |  | |  | |  | | |  |  |
| Depression  No  Yes | Girma et al. (2021) | | | 13-19, 16.83 & 1.3 | 28 | | |  | 121  86 | | | 149  58 | | -28  28 | | .18.841^c^ | | | 1 |  |
| Table 3d | | | | | | | |  |  | | |  | |  | |  | | |  |  |
|  | | Testing study | Subjects Age  Range, mean & SD | | | N | Observed score Mean (SD) | | | Test value  Mean (SD) | Mean difference | | T | | Df | | Cohen’s d | p | | |
| Anxiety & depression | | Perkins & Alo’s (2021) | 8-15, 12.11 & 2.35 | | | 250 | 33.54(14.1) | | | 28.2(13.5) | 5.34 | | 5.442 | | 206 | | .38 | <.001 | | |
| Anxiety | |  | 8-15, 12.11 & 2.35 | | | 250 | 20.52(8.20) | | | 16.8(8.3) | 3.72 | | 6.517 | | 206 | | .45 | <.001 | | |
| Depression | |  | 8-15, 12.11 & 2.35 | | | 250 | 13.70(8.16) | | | 11.3(6.1) | 1.87 | | 3.305 | | 206 | | .23 | .001 | | |

a. 0 cells (0.0%) have expected frequencies less than 5. The minimum expected cell frequency is 68.9.

b. 0 cells (0.0%) have expected frequencies less than 5. The minimum expected cell frequency is 85.9.

c. 0 cells (0.0%) have expected frequencies less than 5. The minimum expected cell frequency is 58.

On the other hand, studies on adolescents' depression symptoms reported approximately equal prevalence rates in Ethiopia, for instance, 36% (29), 38% in Ethiopia (30).

Compared to the Sub-Sahara African adolescents 27% (19), Kenyan adolescents in settlement center, 9.2% (21), in the USA 18% (31), the level of depression in our study was pretty higher. On the other hand, reports that was based on specific population of females in Saudi Arabia at 41.5% (23), and boarding schoolers in Malaysia at 40% (24) found to have similar magnitude. Overall, we found that depression symptoms among adolescents in our study were fairly high and require further attention.

In addition, the level of anxiety and depression among adolescents in our study showed markedly higher anxiety and depression than adolescents in Syrian samples (28). As to depression, it was believed to reach its peak level during such time of adolescence (32) or at least doubled during adolescence from the rate when it was during late childhood (33). Hence, it was supposed to be higher given at least the participants’ age (i.e., 16.53 (SD=1.75) > 12.11 (SD=2.35) see SM D Table 4d) in our study. This study included mid and late-adolescent participants; the Syrian study covered late childhood and early adolescence. According to Kwong et al. (34) depression symptoms get steeper from the age of 11 and through the teenage to the age of twenty and eventually begin decreasing. Looking at the developmental trajectories of anxiety, given the diversified nature of the dimensions, it has a decreasing tendency from infancy to early adolescence (35,36) followed by an increase in anxiety throughout middle and late adolescence (37,38). This could explain why our participants were more anxious than the Syrian samples.

**Supplemental Material (SM) E**

**Do Gender and Family Structure Produce Significant Main and Interaction Effects on Adolescents’ Anxiety and Depression?**

A statistically significant main effect was observed for family structure: F (1,203) = 18.128, p< .001; with more child maltreatment exposure occurring in non-intact families compared to intact families. However, the effect size was medium (partial eta square = .082). No further main effects (gender) or interaction effects were found (main effect gender: F (1, 203) =2.659, p= .105; interaction effect gender*family structure, F (1,203) = .083, p =.363).

The results of this study revealed intriguing insights into the relationship between gender, family structure, anxiety, and depression among adolescents. Surprisingly, the analysis indicated that gender did not exert a significant effect on anxiety and depression, either as a main factor or when considered in the context of family structure. However, it is important to note that a less stringent test, specifically correlation analysis, did reveal a small yet significant correlation (r = .144, p < .05) between gender and symptoms of anxiety and depression.

This finding stands in contrast to previous research on gender differences in anxiety and depression. Studies by de Lijster et al. (36) and Van Oort et al. (37) reported that females tended to exhibit a higher proportion of anxiety symptoms, while research conducted by Tareke et al. (30) and Tirfeneh & Srahbzu (29) indicated that females experienced more depressive symptoms than males. Moreover, the interplay between gender differences and family environment was highlighted in studies such as Wang et al. (39), which suggested that gender disparities in anxiety and depression could be further accentuated based on the specific family context in which adolescents were raised.

Further complicating the picture, Assari et al. (40) found that adolescents growing up in intact married parents' families at the age of 15 were associated with fewer anxiety symptoms, providing a protective factor against elevated anxiety levels when compared to their counterparts in non-intact families.

These diverse findings underscore the complexity of the relationship between gender, family structure, and mental health outcomes among adolescents. Future research should continue to explore these factors in depth, considering nuanced variables within family dynamics, to gain a comprehensive understanding of the intricate interplay between gender, family context, and the prevalence of anxiety and depression among young individuals.

In a study conducted by Fung (41) in China, it was highlighted that adolescents living with their stepmothers exhibited the highest symptoms of anxiety and depression, irrespective of their gender. Fung's research delved into the complexities of blended families, suggesting that non-biological mothers face greater challenges in establishing emotional connections with adolescents at home compared to biological mothers. This difficulty in bonding could be attributed to various factors, such as differences in upbringing, personal dynamics, and role expectations within the family unit.

Moreover, Dawson (13) further supported the notion that family structure significantly impacts adolescents' mental health. According to Dawson's findings, children residing in single-parent households or with mothers and stepfathers were more prone to developing behavioral problems compared to those living with both biological parents. This negative effect on mental health could stem from several factors prevalent in non-intact families, including reduced parental support, heightened responsibilities, limited control over their environment, increased punitive measures, elevated sibling conflicts, and diminished family cohesion, as highlighted by Amato's research in 1987 (42).

These insights collectively emphasize the intricate interplay between family structure, parental relationships, and adolescents' mental well-being. Understanding these factors is crucial for implementing targeted interventions and support systems that address the specific challenges faced by adolescents in non-traditional family setups.

SM E Table 5*:*

*Males’ and Females’ Anxiety and Depression Scores by Family Structures* and the ANOVA Summary of the Effects of Family Structures and Gender

| **Variables** | **Child maltreatment** | | | | | |  | **Type II sum of squares** | **df** | | **Mean square** | **F** | **Sig.** | **Partial Eta Squares** |
| --- | --- | --- | --- | --- | --- | --- | --- | --- | --- | --- | --- | --- | --- | --- |
| **Gender** | **Intact** | | | **Non-intact** | | | **Source** |  |  | |  |  |  |  |
|  | **N** | **Mean** | **SD** | **N** | **Mean** | **SD** |  |  |  | |  |  |  |  |
| Male | 43 | 27.86 | 13.40 | 50 | 34.24 | 13.82 | Corrected Model | 4392.028a | 3 | | 1464.009 | 8.119 | <.001 | .107 |
| Female | 43 | 29.23 | 13.41 | 71 | 39.08 | 13.81 | Intercept | 211036.574 | 1 | | 211036.574 | 1170.329 | <.001 | .852 |
| Total | 86 | 28.55 | 13.34 | 121 | 37.08 | 13.60 | Gender | 479.503 | 1 | | 479.503 | 2.659 | .105 | .013 |
|  |  |  |  |  |  |  | Family structure | 3268.904 | 1 | | 3268.904 | 18.128 | <.001 | .082 |
|  |  |  |  |  |  |  | Gender * Fam_stru | 149.606 | 1 | | 149.606 | .830 | .363 | .004 |
|  |  |  |  |  |  |  | Error | 36605.450 | 203 | | 180.322 |  |  |  |
|  |  |  |  |  |  |  | Total | 273806.000 | 207 | |  |  |  |  |
|  |  |  |  |  |  |  | Corrected Total | 40997.478 | 206 | |  |  |  |  |
| a. R Squared = .107 (Adjusted R Squared = .094) | | | | | | | | | |  |  |  |  |  |

**Supplemental Material (SM) F**

**Parents’ Level of Psychological Distress (PD)**:

In order to assess the level of current psychological distress experienced by parental participants, a one-sample t-test was administered. In the present investigation, we examined the mean current psychological distress ratings among parents compared to the normative data provided for adults in the (43) study conducted in the Australian context. Although there exist chronological and societal distinctions between the two populations, conducting a comparison would potentially provide valuable insight into our study's discoveries. There was a noteworthy distinction in the degree of psychological distress as demonstrated by statistical analysis (t _(183)_ = 22.854, p.001). Based on the results, it has been established that the current sample exhibits a higher level of anxiety in comparison to the Australian sample of 2001. The present study has revealed a statistically significant and notable magnitude of effect, as Cohen's d was observed to have a sizeable value of 1.685 in the practical significance analysis.

In addition, a useful comparison was made on the proportion of parents who suffered from psychological distress with another study in Ethiopia (44,45) and Tanzania (46). The Chi-square goodness of fit test was conducted for the same purpose and found that there was a statistically significant difference between the number of participants with psychological distress in this study (43.48%) and working adults in Addis Abeba (17.7%) (45), (X^2^ (184) = 190.368, p<.001). The findings showed that individuals in this research suffered much more than participants in the same setting in 2012. However, no statistically significant difference was found against healthcare professionals in Gondar (44.4%), (X^2^ (184) = .063, p=.801). Although not statistically significant, the result shows a slightly lower proportion of participants suffered in this study than in the Gondar sample (44). However, the same comparison of our participants (28.80% of likely well, 27.72% of Mild PD, 32.07% of moderate PD, and 11.41% of Severe PD) with the Tanzanian sample (78.4% of likely well, 13.4% of Mild PD, 5.7% of moderate PD and 2.6% of Severe PD) (46) revealed a statistically significant difference, (X^2^ _(184)_ = 365.598, p<.001). This suggests a larger proportion of participants in this study were found to suffer from PD than the Tanzanian sample.

SM F Table 6, the level of Parents’ Current Psychological Distress (N=184)

|  | | Mean (SD) | | Test Value  Mean (SD) | | Mean difference | | T | df | | Cohen’s d | | p |
| --- | --- | --- | --- | --- | --- | --- | --- | --- | --- | --- | --- | --- | --- |
|  | |  | |  | |  | |  |  | |  | |  |
| Psychological Distress against Andrews & Slade (2001) | | 24.12(5.89) | | 14.2 | | 9.92 | | 22.854 | 183 | | 1.685 | | <.001 |
| Variable | Observed | | Expected | | Residual | | X^2^ | | | DF | | Assy. Sig | |
| Current PD- against Kabito et al.  No  Yes | 104  80 | | 102.3  81.7 | | 1.7  -1.7 | | .063^a^ | | | 1 | | .801 | |
| Current PD- against Gelaye et al.  No  Yes | 104  80 | | 32.6  151.4 | | 71.4  -71.4 | | 190.368^b^ | | | 1 | | <.001 | |
| Current PD- against Ivanova et al.  Well | 53 | | 114.1 | | -91.1 | | 365.598^c^ | | | 3 | | <.001 | |
| Mild | 51 | | 24.6 | | 26.4 | |  | | |  | |  | |
| Moderate | 59 | | 10.5 | | 48.5 | |  | | |  | |  | |
| Severe | 21 | | 4.8 | | 16.2 | |  | | |  | |  | |

| 1. a 0 cells (0.0%) have expected frequencies less than 5. The minimum expected cell frequency is 81.7. 2. b 0 cells (0.0%) have expected frequencies less than 5. The minimum expected cell frequency is 32.6. 3. c 1 cells (25.0%) have expected frequencies less than 5. The minimum expected cell frequency is 4.8. |
| --- |

According to the findings of this study, the degree of psychological distress in the parent participants was substantially greater than the normative data reported by Andrews & Slade, (43) in the Australian sample. Given the societal and economic diversity, this may not be a fair comparison of two diverse groups. With this comparison, we also compared the number of parents with psychological distress in this study to another local study done in the same context, which revealed a substantially greater proportion of participants with psychological distress than Gelaye et al., (45) study. However, the time gap between the two studies and the metrics utilized in the two research might explain the disparity. As Ethiopia is experiencing the worst humanitarian crisis in decades (47), as well as an economic situation that will be influenced in some way by the current global and local situations, it should expound on the participants' psychological situation in comparison to the country's relatively stable situation in 2012.

However, when compared to recent research in Gondar, the ratio of participants with psychological distress in our study did not differ substantially from Kabito and Mekonnen's (44) study. Despite the fact that the two cities are more than 700 kilometers apart, the fact that both studies were done in the post-COVID-19 era and under dangerous socio-political and economic conditions makes the results comparable. We included an additional comparison to a Tanzanian sample to provide a comparative picture of the prevalence rate of psychological distress in our partakers. The findings of this investigation revealed that many more people in our study were found to have psychological distress than participants in the Tanzanian sample (46). This is most likely due to the present scenario in Ethiopia. As regards gender, this study found no statistically significant difference between males and females in psychological distress. In contrast to our findings, previous studies reported that females had much more psychological than their male counterparts (45,46).

**Supplemental Material (SM) G**

**Exploring the Gender-Specific Cascade: Parental History, Adolescent Maltreatment, and Anxiety-Depression Pathways under Psychological Distress**

Even though the number of father and mother participants was too small to do this conditional analysis from father-to-adolescents and mother-to -adolescents cascade, our preliminary exploration seems that the moderation mediation was likely driven by fathers.

**SM G Table 7,**

**Direct and indirect effects of the moderated mediation model**

| Direct relationships/pathways | | Mothers (n=95)  Fathers (n=89) | | | Unstandardized  Coefficient | | | t values | | p value | |
| --- | --- | --- | --- | --- | --- | --- | --- | --- | --- | --- | --- |
| History of CAN 🡪 CM (a-path) | | Mothers  Fathers | | | 7.501  8.057 | | | 1.210  1.760 | | 0.229  0.082 |  |
| CM 🡪 Anxiety & Depression (b-path) | | Mothers  Fathers | | | 0.224  0.237 | | | 2.955  3.674 | | 0.004  <0.001 |  |
| History of CAN 🡪 Anxiety & Depression (c’-path) | | Mothers  Fathers | | | 5.296  7.534 | | | 1.897  2.967 | | 0.061  0.004 |  |
| History of CM*PD 🡪 CM | | Mothers  Fathers | | | -0.990  -2.418 | | | -0.738  -2.299 | | 0.462  0.024 |  |
| Conditional indirect effects of Parents history of CAN on Adolescents’ anxiety and depression:  Parents’ CAN -> CM -> Adolescents’ Anxiety and Depression | | | | | | | | | | |  |
| Different levels parents’ PD | | | Effect | BootSE | | t value | BootLLCI | | BootULCI | |  |
| Mothers (n=95)  Fathers (n=89) | -6.188  -5.457 | | 3.056  5.079 | 1.676  1.690 | | 1.823  3.005 | -0.094  1.920 | | 6.534  8.552 | |  |
| Mothers (n=95)  Fathers (n=89) | .000  .000 | | 1.682  1.906 | 1.566  1.269 | | 1.074  1.502 | -.826  -0.299 | | 5.453  4.782 | |  |
| Mothers (n=95)  Fathers (n=89) | 6.188  5.457 | | -0.309  -1.267 | 2.943  1.812 | | 0.105  0.699 | -6.087  -4.536 | | 7.775  2.890 | |  |
| Index of moderated mediation: | | | Index | BootSE | | t value | BootLLCI | | BootULCI | |  |
| Current psychological distress | Mothers  Fathers | | -0.222  -0.572 | 0.293  0.218 | | 0.758  2.624 | -0.730  -4.536 | | 0.475  -0.113 | |  |

Note. CAN = Child abuse and neglect experiences in parents as children, PD = parental current psychological distress, CM = child maltreatment experiences of adolescents

Reference

1. Bede A. Prevalence and Nature of Child Neglect and Mental Health Status of Secondary School Adolescents. Adv Soc Sci Res J. 2015 Apr 25;2(4).

2. Gallo EAG, Munhoz TN, Loret de Mola C, Murray J. Gender differences in the effects of childhood maltreatment on adult depression and anxiety: A systematic review and meta-analysis. Vol. 79, Child Abuse and Neglect. Elsevier Ltd; 2018. p. 107–14.

3. Asnake M. Victimization and Mental Health Problems of Children and Adolescents in Gondar Town, North Western Ethiopia. [Addis Ababa]: Addis Ababa University; 2015.

4. Liao M, Lee AS, Roberts-Lewis AC, Hong JS, Jiao K. Child maltreatment in China: An ecological review of the literature. Child Youth Serv Rev. 2011 Sep;33(9):1709–19.

5. Mulatie M. Physical and Psychological Child Abuse in Ethiopia: Implications for Intervention. J Psychol Psychother. 2014;04(02).

6. Meinck F, Cluver LD, Boyes ME, Loening-Voysey H. Physical, emotional and sexual adolescent abuse victimisation in South Africa: prevalence, incidence, perpetrators and locations. J Epidemiol Community Health [Internet]. 2016 [cited 2022 Mar 2];70. Available from: http://dx.doi.org/10.1136/jech-2015-205860

7. Lee J, Kim H, Chang SM, Hong JP, Lee DW, Hahm BJ, et al. The Association of Childhood Maltreatment with Adulthood Mental Disorders and Suicidality in Korea: a Nationwide Community Study. J Korean Med Sci. 2021;36(37):1–14.

8. Meng X, D’Arcy C. Gender moderates the relationship between childhood abuse and internalizing and substance use disorders later in life: A cross-sectional analysis. BMC Psychiatry. 2016 Nov 15;16(1).

9. Bras H, Mandemakers J. Maternal education and sibling inequalities in child nutritional status in Ethiopia. SSM Popul Health. 2022 Mar 1;17.

10. Abera L, Aliye A, Tadesse K, Guta A. Magnitude of child sexual abuse and its associated factors among high school female students in Dire Dawa, Eastern Ethiopia: a cross-sectional study. Reprod Health. 2021 Dec 1;18(1).

11. Deyessa N, Berhane Y, Ellsberg M, Emmelin M, Kullgren G, Högberg U. Violence against women in relation to literacy and area of residence in Ethiopia. Glob Health Action. 2010 Dec;3(1):2070.

12. Pankhurst A, Negussie N, Mulugeta E. Understanding Children’s Experiences of Violence in Ethiopia: Evidence from Young Lives [Internet]. 2016. Available from: www.unicef-irc.org

13. Dawson DA. Family Structure and Children’s Health and Well-Being: Data from the 1988 National Health Interview Survey on Child Health. Journal of Marriage and Family [Internet]. 1991;53(3):573–84. Available from: https://www.jstor.org/stable/352734

14. Jackson S, Thompson RA, Christiansen EH, Colman RA, Wyatt J, Buckendahl CW, et al. PREDICTING ABUSE-PRONE PARENTAL ATTITUDES AND DISCIPLINE PRACTICES IN A NATIONALLY REPRESENTATIVE SAMPLE. Vol. 23, Child Abuse & Neglect. 1999.

15. Debowska A, Hales G, Boduszek D. Violence against children by stepparents.

16. Nakie G, Segon T, Melkam M, Desalegn GT, Zeleke TA. Prevalence and associated factors of depression, anxiety, and stress among high school students in, Northwest Ethiopia, 2021. BMC Psychiatry. 2022 Dec 1;22(1).

17. Berhanu Boru B, Yonas Deressa G. A Systematic Review and Meta-Analysis of Anxiety among Children and Youth in Ethiopia. Journal of Depression and Anxiety Disorders. 2021 Jul 5;3(2).

18. Abebe Gebreegziabher Z, Eristu R, Molla A. Depression, Anxiety, Somatic symptom and their determinants among High School and Preparatory School Adolescents in Gondar Town, Northwest Ethiopia, 2022.Non-recursive Structural Equation Modeling. Available from: https://doi.org/10.1101/2023.01.27.23285096

19. Jorns-Presentati A, Napp AK, Dessauvagie AS, Stein DJ, Jonker D, Breet E, et al. The prevalence of mental health problems in sub-Saharan adolescents: A systematic review. PLoS One. 2021 May 1;16(5 May).

20. Kuringe E, Materu J, Nyato D, Majani E, Ngeni F, Shao A, et al. Prevalence and correlates of depression and anxiety symptoms among out-of-school adolescent girls and young women in Tanzania: A cross-sectional study. PLoS One. 2019 Aug 1;14(8).

21. Friedberg R, Baiocchi M, Rosenman E, Amuyunzu-Nyamongo M, Nyairo G, Sarnquist C. Mental health and gender-based violence: An exploration of depression, PTSD, and anxiety among adolescents in Kenyan informal settlements participating in an empowerment intervention. PLoS One. 2023 Mar 1;18(3 March).

22. Frank-Briggs AI, Alikor EAD. Anxiety Disorder amongst Secondary School Children in an Urban City in Nigeria [Internet]. Vol. 6, Int J Biomed Sci. 2010. Available from: www.ijbs.org

23. Al-Gelban KS, Al-Amri HS, Mostafa OA. Prevalence of Depression, Anxiety and Stress as Measured by the Depression, Anxiety, and Stress Scale (DASS-42) among Secondary School Girls in Abha, Saudi Arabia. Vol. 9. Epub; 2009.

24. Wahab S, Rahman FNA, Wan Hasan WMH, Zamani IZ, Arbaiei NC, Khor SL, et al. Stressors in secondary boarding school students: Association with stress, anxiety and depressive symptoms. Asia-Pacific Psychiatry. 2013 Apr;5(SUPPL. 1):82–9.

25. Girma S, Tsehay M, Mamaru A, Abera M. Depression and its determinants among adolescents in Jimma town, Southwest Ethiopia. PLoS One. 2021 May 1;16(5 May).

26. Senait S. Prevalence of Depression among Adolescents and Association of Parental Neglect on Depression in Governmental Preparatory Schools in Addis Ababa, Ethiopia. [Addis Ababa]: Addis Ababa University; 2017.

27. Chekol AT, Wale MA, Abate AW, Beo EA, Said EA, Negash BT. Predictors of depression among school adolescents in Northwest, Ethiopia, 2022: institutional based cross-sectional. BMC Psychiatry. 2023 Dec 1;23(1).

28. Perkins JD, Alós J. Rapid mental health screening in conflict zones: a translation and cross-cultural adaptation into Arabic of the shortened Revised Child Anxiety and Depression Scale (RCADS-25). Confl Health. 2021 Dec 1;15(1).

29. Tirfeneh E, Srahbzu M. Depression and Its Association with Parental Neglect among Adolescents at Governmental High Schools of Aksum Town, Tigray, Ethiopia, 2019: A Cross Sectional Study. Depress Res Treat. 2020;2020.

30. Tareke SA, Lelisho ME, Hassen SS, Seid AA, Jemal SS, Teshale BM, et al. The Prevalence and Predictors of Depressive, Anxiety, and Stress Symptoms Among Tepi Town Residents During the COVID-19 Pandemic Lockdown in Ethiopia. J Racial Ethn Health Disparities. 2023 Feb 1;10(1):43–55.

31. Saluja G, Iachan R, Scheidt PC, Overpeck MD, Sun W, Giedd JN. Prevalence of and Risk Factors for Depressive Symptoms Among Young Adolescents. Vol. 158, Arch Pediatr Adolesc Med. 2004.

32. Ferro MA, Gorter JW, Boyle MH. Trajectories of Depressive Symptoms in Canadian Emerging Adults. 2015.

33. Jane Costello E, Erkanli A, Angold A. Is there an epidemic of child or adolescent depression? J Child Psychol Psychiatry. 2006 Dec;47(12):1263–71.

34. Kwong ASF, Manley D, Timpson NJ, Pearson RM, Heron J, Sallis H, et al. Identifying Critical Points of Trajectories of Depressive Symptoms from Childhood to Young Adulthood. J Youth Adolesc. 2019 Apr 1;48(4):815–27.

35. Costello EJ, Mustillo S, Erkanli A, Keeler ; Gordon, Angold A. Prevalence and Development of Psychiatric Disorders in Childhood and Adolescence. Arch general psychiatry. 2003;60:837–44.

36. de Lijster JM, van den Dries MA, van der Ende J, Utens EMWJ, Jaddoe VW, Dieleman GC, et al. Developmental Trajectories of Anxiety and Depression Symptoms from Early to Middle Childhood: a Population-Based Cohort Study in the Netherlands. J Abnorm Child Psychol. 2019 Nov 1;47(11):1785–98.

37. Van Oort FVA, Greaves-Lord K, Verhulst FC, Ormel J, Huizink AC. The developmental course of anxiety symptoms during adolescence: The TRAILS study. J Child Psychol Psychiatry. 2009 Oct;50(10):1209–17.

38. Van Oort FVA, Ormel J, Verhulst FC. Angstsymptomen bij adolescenten; bevindingen uit de trails-studie. tijdschrift voor psychiatrie . 2012;54(5):463–9.

39. Wang L, Zhang Y, Yin H, Zhang Z, Tao Y, Xu Y, et al. The effects of parental relationships, and gender and grade differences on depressive disorder in Chinese adolescents: the evidence from multiple cross-sectional surveys (1999–2016). China Popul Dev Stud. 2019 Oct;3(1):37–52.

40. Assari S, Caldwell CH, Zimmerman MA. Family structure and subsequent anxiety symptoms; minorities’ diminished return. Brain Sci. 2018;8(6).

41. Fung ALC. The Significance of Family Structure in Internalizing (Anxious/Depressed) and Externalizing (Aggressive/Delinquent) Problems among Chinese Adolescents. Appl Res Qual Life. 2021 Dec 1;16(6):2403–18.

42. Amato PR. Family Processes in One-Parent, Stepparent, and Intact Families: The Child’s Point of View [Internet]. Vol. 49, Source: Journal of Marriage and Family. 1987. Available from: https://www.jstor.org/stable/352303

43. Andrews G, Slade T. Interpreting scores on the Kessler Psychological Distress Scale (K10). Aust N Z J Public Health. 2001;25(6):494–7.

44. Kabito GG, Mekonnen TH. Psychological distress symptoms among healthcare professionals are significantly influenced by psychosocial work context, Ethiopia: A cross-sectional analysis. PLoS One. 2020 Sep 1;15(9 September).

45. Gelaye B, Lemma S, Deyassa N, Bahretibeb Y, Tesfaye M, Berhane Y, et al. Prevalence and Correlates of Mental Distress Among Working Adults in Ethiopia. Vol. 8. 2012.

46. Ivanova O, Sineke T, Wenzel R, Siyame E, Lalashowi J, Bakuli A, et al. Health-related quality of life and psychological distress among adults in Tanzania: a cross-sectional study. Archives of Public Health. 2022 Dec 1;80(1).

47. Lima NNR, de Moura Gabriel IW, Pires JP, Neto JC, da Silva JIM, Júnior JRM, et al. Abuse and neglect among Ethiopian children and adolescents. Vol. 127, Child Abuse and Neglect. Elsevier Ltd; 2022.
